# An integrated quantification method to increase the precision, robustness, and resolution of protein measurement in human plasma samples

**DOI:** 10.1186/1559-0275-12-3

**Published:** 2015-01-29

**Authors:** Xiao-jun Li, Lik Wee Lee, Clive Hayward, Mi-Youn Brusniak, Pui-Yee Fong, Matthew McLean, JoAnne Mulligan, Douglas Spicer, Kenneth C Fang, Stephen W Hunsucker, Paul Kearney

**Affiliations:** Integrated Diagnostics, 219 Terry Avenue North, Suite 100, 98109 Seattle, WA USA; Fred Hutchinson Cancer Research Center, 1100 Fairview Ave N., M4-A830, 98109 Seattle, WA USA; DuPont Industrial Biosciences, 925 Page Mill Road, Palo, 94304 Alto, CA USA

**Keywords:** Multiple reaction monitoring, Plasma or serum analysis, Quantitative proteomics, Clinical proteomics, Mass spectrometry, Immunoaffinity depletion, Bioinformatics

## Abstract

**Background:**

Current quantification methods for mass spectrometry (MS)-based proteomics either do not provide sufficient control of variability or are difficult to implement for routine clinical testing.

**Results:**

We present here an integrated quantification (InteQuan) method that better controls pre-analytical and analytical variability than the popular quantification method using stable isotope-labeled standard peptides (SISQuan). We quantified 16 lung cancer biomarker candidates in human plasma samples in three assessment studies, using immunoaffinity depletion coupled with multiple reaction monitoring (MRM) MS. InteQuan outperformed SISQuan in precision in all three studies and tolerated a two-fold difference in sample loading. The three studies lasted over six months and encountered major changes in experimental settings. Nevertheless, plasma proteins in low ng/ml to low μg/ml concentrations were measured with a median technical coefficient of variation (CV) of 11.9% using InteQuan. The corresponding median CV using SISQuan was 15.3% after linear fitting. Furthermore, InteQuan surpassed SISQuan in measuring biological difference among clinical samples and in distinguishing benign versus cancer plasma samples.

**Conclusions:**

We demonstrated that InteQuan is a simple yet robust quantification method for MS-based quantitative proteomics, especially for applications in biomarker research and in routine clinical testing.

**Electronic supplementary material:**

The online version of this article (doi:10.1186/1559-0275-12-3) contains supplementary material, which is available to authorized users.

## Background

**Figure 1:**
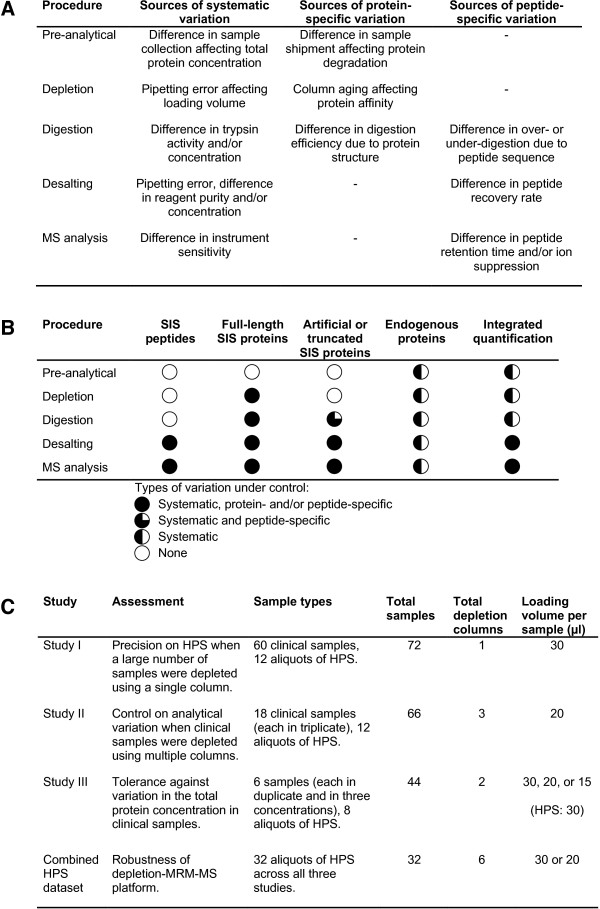
**Overview of experimental variations and control methods. (A)** Exemplar sources of variations. Systematic variations affect all proteins or peptides similarly. Protein- or peptide-specific variations affect only particular proteins or peptides. Random variations are not listed because they are not controllable. **(B)** Control of variations by different quantification methods in the analysis of plasma samples on a depletion-MRM-MS platform. **(C)** Overview of the three assessment studies and the combined HPS dataset.

Multiple reaction monitoring (MRM, also known as selected reaction monitoring) mass spectrometry (MS) allows for the fast and reproducible measurement of tens to hundreds of proteins in complex biological samples such as bio-fluids, tissues, and cultured cells [1–5]. There is tremendous interest in applying the technology to develop blood-based clinical tests for the diagnosis, prognosis or treatment stratification of various diseases [6–9]. Due to the high complexity of the human blood proteome [10], proteomic analysis of blood samples (that is serum or plasma) typically consists of multiple experimental steps and is prone to variation [1, 11] (Figure 1A). In addition, changes in laboratory conditions (e.g., operators, instruments, reagents) are expected during routine laboratory operations in clinical testing. Therefore, controlling analytical variability to satisfy rigorous quality control requirements for blood-based clinical testing using MRM-MS platforms has been challenging.

The principle of stable isotope labeling (SIL) is widely used in MS-based quantitative proteomics to control experimental variability [12–18]. Protein abundance is measured by comparing MS signal intensities of endogenous peptides with those of their corresponding stable isotope-labeled internal standard (SIS) peptides. Three SIL approaches are potentially suitable for clinical testing (Figure 1B). The first approach utilizes SIS peptides for protein quantification [12, 13] (SISQuan) and is the simplest one for implementation. SIS peptides are synthesized, optimized for MS analysis and spiked into samples before [12] or after [13] protein digestion to control variation in post-digestion procedures. However, variation occurring before or during digestion is not controlled. The second approach [14–16] spikes full-length SIS proteins into samples before any analytical procedure takes place. While this approach offers the best control of analytical variability, it is applicable only to soluble proteins. Quality control of the production, the storage, etc., of SIS proteins as standards is challenging for routine laboratory operations [14]. The third approach spikes either artificial [17] or truncated [18] SIS proteins into samples before protein digestion. It controls most variation in protein digestion and variation in subsequent procedures. However, it cannot control variation occurring before digestion and faces similar implementation challenges as the second approach. None of the above SIL approaches can control pre-analytical variability associated with sample collection and handling. A simple and robust method that provides sufficient control of pre-analytical and analytical variability for routine clinical testing on MS-based proteomics platforms is still lacking.

We recently analyzed hundreds of human plasma samples in a discovery study of lung cancer biomarkers [6], using immunoaffinity-based protein depletion coupled with MRM-MS (depletion-MRM-MS). In the study we identified six endogenous normalizing proteins from 371 protein candidates. Since the normalizing proteins were processed and analyzed together with target proteins of interest, we expected them to serve as monitors for systematic variation in both pre-analytical and analytical procedures (Figure 1B). We verified that experimental variability was reduced after normalization by a panel of the six proteins. Although this method of endogenous protein normalization (EPN) and similar approaches have been reported previously in quantitative proteomics [7, 19–22], our approach is unique. The six normalizing proteins were selected by their ability to compensate both the drift of depletion columns and the technical variation of other proteins [6], rather than their ‘housekeeping" properties as utilized in other approaches. However, the EPN quantification method used in our previous study is a label-free approach and cannot control analytical variability as narrowly as the three SIL approaches.

Since our label-free discovery study [6], we have developed more accurate MRM assays for target proteins of interest [23], using the SIL approach of SIS peptides. As reviewed above, neither SIL nor EPN is ideal for protein quantification in clinical proteomics. To deal with this challenge, we present here a new quantification method, named integrated quantification (InteQuan), to combine the advantageous features of the two methods: The six endogenous normalizing proteins were used to compensate systematic variation in pre-analytical procedures and in depletion and digestion; SIS peptides were used to control variation in desalting and MS analysis (Figure 1B). To mimic an actual clinical testing scenario, we completely defined details of InteQuan method based on data from a different study [23] before assessing its performance in three independent studies (Figure 1C). To the best of our knowledge, no one has described such a method to quantify individual proteins before. A recent work used "sparse reference labeling" to anchor protein abundance that can be valuable for biomarker discovery [24]. Nevertheless, individual proteins were essentially quantified in a label-free approach in the study, leaving peptide-specific variation in MS analysis uncontrolled and thus reducing its validity for routine clinical testing. In this study we demonstrated that InteQuan increased the precision, robustness, and resolution of protein measurement in the three independent assessment studies.

## Results and discussion

### Protein quantification in human plasma samples

A total of 21 lung cancer biomarker candidates were identified in our recent discovery study [6]. Two of the 21 candidates (GSLG1 and EF1A1) were eliminated from this study due to weak signals on a new MRM-MS platform. Another candidate (FIBA) was eliminated due to its affinity to the depletion column [25]. The remaining 18 candidates (Table 1) were targeted for quantification in human plasma samples.

**Table 1:** **List of six normalizing proteins and eighteen target proteins of interest**

Protein (HUMAN)	Protein name	Concentration ^***a***^ (ng/ml)	Transition ^***b***^ (peptide_Q1_Q3)	FDR ^***c***^	***F***_***n***_^***d***^	^***e***^	***Ă***_***n***_^***f***^
*Normalizing proteins*							
PEDF	Pigment epithelium-derived factor	7200	LQSLFDSPDFSK_692.34_593.30	1.40E-04	0.971	1.756	1.209E06
MASP1	Mannan-binding lectin serine protease 1	240	TGVITSPDFPNPYPK_816.92_258.10	5.75E-04	0.957	0.360	1.060E05
GELS	Gelsolin	16000	TASDFITK_441.73_710.40	3.18E-04	0.852	0.502	1.897E06
LUM	Lumican	4000	SLEDLQLTHNK_433.23_499.30	3.82E-04	0.838	10.846	4.717E06
C163A	Scavenger receptor cysteine-rich type 1 protein M130	94	INPASLDK_429.24_630.30	1.19E-03	0.823	0.392	4.690E04
PTPRJ	Receptor-type tyrosine-protein phosphatase eta	9.9	VITEPIPVSDLR_669.89_896.50	1.44E-03	0.926	0.275	4.685E04
*Target proteins of interest*							
AIFM1	Apoptosis-inducing factor 1, mitochondrial	1.4	ELWFSDDPNVTK_725.85_558.30	3.70E-02	Assay specificity not verified		
KIT	Mast/stem cell growth factor receptor	8.2	YVSELHLTR_373.21_428.30	2.40E-03	0.730		
FRIL	Ferritin light chain	12	LGGPEAGLGEYLFER_804.40_1083.60	4.30E-05	0.844		
LRP1	Prolow-density lipoprotein receptor-related protein 1	20	TVLWPNGLSLDIPAGR_855.00_1209.70	1.40E-04	Assay specificity not verified		
COIA1	Collagen alpha-1(XVIII) chain	35	AVGLAGTFR_446.26_721.40	6.70E-04	0.732		
PRDX1	Peroxiredoxin-1	60	QITVNDLPVGR_606.30_970.50	1.90E-05	1.714		
TENX	Tenascin-X	70	YEVTVVSVR_526.29_293.10	1.10E-03	0.699		
ENPL	Endoplasmin	88	SGYLLPDTK_497.27_308.10	1.10E-03	0.649		
GRP78	78 kDa glucose-regulated protein	100	TWNDPSVQQDIK_715.85_288.10	1.80E-03	1.140		
BGH3	Transforming growth factor-beta-induced protein ig-h3	140	LTLLAPLNSVFK_658.40_804.50	1.40E-04	0.779		
ALDOA	Fructose-bisphosphate aldolase A	250	ALQASALK_401.25_617.40	3.70E-05	0.777		
GGH	Gamma-glutamyl hydrolase	250	YYIAASYVK_539.28_638.40	1.70E-03	0.834		
CD14	Monocyte differentiation antigen CD14	420	ATVNPSAPR_456.80_527.30	4.30E-04	0.789		
LG3BP	Galectin-3-binding protein	440	VEIFYR_413.73_598.30	2.80E-05	0.842		
TSP1	Thrombospondin-1	510	GFLLLASLR_495.31_559.40	1.90E-05	0.625		
IBP3	Insulin-like growth factor-binding protein 3	5700	FLNVLSPR_473.28_685.40	2.80E-05	0.790		
TETN	Tetranectin	58000	LDTLAQEVALLK_657.39_871.50	3.70E-05	0.760		
ISLR	Immunoglobulin superfamily containing leucine-rich repeat protein		ALPGTPVASSQPR_640.85_841.50	4.40E-03	0.850		

^*a*^Predicted plasma concentration [26]. ^*b*^The transition that was used for quantification. ^*c*^False discovery rate for peptide MRM assay (peptide Q value) [6]. ^*d*^Correction factor {*F*_*n*_} in Study II in which a new lot of SIS peptides were used. ^*e*^Scaling constant  for InteQuan. ^*f*^Scaling constant {*Ă*_*n*_} for EPN

Six endogenous normalizing proteins (Table 1) were selected from a pool of 371 protein candidates in our previous label-free discovery study [6]. The predicted plasma concentration [26] of the six proteins, estimated from the occurrence of protein detection in human plasma or serum samples by the proteomics community, ranged from 9.9 ng/ml (PTPRJ) to 16 μg/ml (GELS). All six proteins were used as normalizing proteins for InteQuan and for EPN in this study.

Human plasma samples were analyzed on a depletion-MRM-MS platform. SIS peptides of the target and the normalizing proteins were synthesized and spiked into peptide samples after digestion. The specificity of MRM assays to the corresponding proteins was verified for all proteins except for LRP1 and AIFM1. As shown in Additional file 1: Figure S1, MRM signals of verified assays were well above the corresponding noise level; endogenous and SIS peptides co-eluted and had comparable intensity ratios between different transitions. The highest false discovery rate (FDR) of the original assays was 0.44% (ISLR, see Table 1). As shown later in Study III, proteins were also measured within the respective linear dynamic range of the assays. Two blank samples were processed and analyzed at the end of each experimental batch in Study II and III to monitor possible carryover from previous samples (see Additional file 2: Table S1). MRM signals in those blank samples were just above noise level (data not shown), indicating that carryover was not a problem for the depletion-MRM-MS platform. After validating the MRM assays, LRP1 and AIFM1 were both eliminated from further analysis. The predicted plasma concentration [26] of the 16 remaining target proteins spanned four orders of magnitude from 8.2 ng/ml (KIT) to 58 μg/ml (TETN).

The target proteins were quantified based on MRM-MS data using four different methods (raw MS data, EPN, SISQuan and InteQuan). In this study, the abundance of a protein was evaluated based on the MRM signal intensity of the strongest transition from the protein, as previously justified [2, 6]. Thus, no distinction was made between protein abundance, peptide abundance and transition abundance. For raw MS data, protein abundance was measured by the peak area of the strongest transition of the protein. For EPN, protein abundance using the raw MS data was divided by a sample-dependent normalization factor that was calculated from the peak areas of the six normalizing proteins. Six scaling constants, one for each of the six normalizing proteins, were used in the calculation of the normalization factor. For SISQuan, protein abundance was measured by the response ratio between the peak area of the strongest transition of the target protein and the peak area of the matching transition of the corresponding SIS peptide. For InteQuan, protein abundance using SISQuan was divided by a sample-dependent normalization factor that was calculated from the response ratios of the six normalizing proteins. As with EPN, six scaling constants were used in the calculation of the normalization factor. In the study, we mainly focused on comparing the new InteQuan method with the widely used SISQuan method.

All scaling constants for InteQuan and for EPN (Table 1) were determined from a different study [23] of 100 clinical samples and 20 aliquots of a human plasma standard (HPS) sample. None of the scaling constants were modified in this study. Therefore, the assessment of the four quantification methods was based on independent datasets.

### Demonstration of complementary control of variation

In Study I, 60 clinical samples and 12 aliquots of the HPS sample were analyzed in three experimental batches using one depletion column (Figure 1C and Additional file 2: Table S1). Clinical information of the patients is listed in Additional file 3: Table S2. MRM-MS data was successfully collected on 55 clinical samples and 10 HPS samples while seven samples were lost during processing (Additional file 2: Table S1). The normalization factors of the six normalizing proteins had a median coefficient of variation (CV) of 20.4% as evaluated from individual samples in the study.

**Figure 2:**
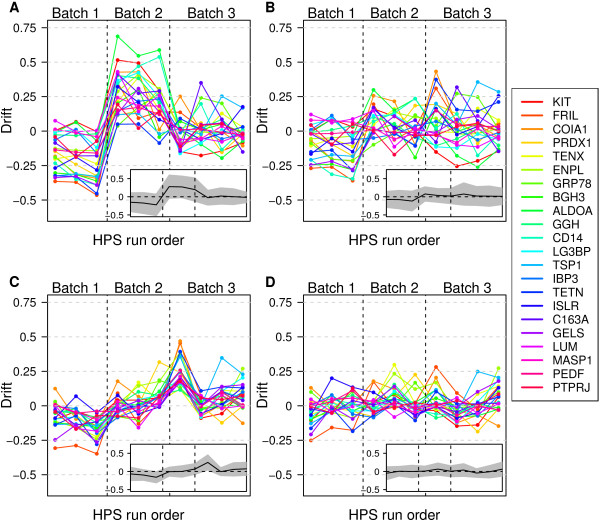
**Intensity drift of the 16 target proteins and the six normalizing proteins as measured on the 10 HPS samples in Study I.** Results were obtained for **(A)** raw MS data, **(B)** EPN, **(C)** SISQuan, or **(D)** InteQuan. Insert: mean (solid line) and 95% confidence interval (shaded band) of protein drifts.

To understand how SISQuan and EPN controlled technical variability, intensity drift was defined as the relative deviation of protein intensity in individual samples from the corresponding median intensity in all samples, and was evaluated based on data of the 10 HPS samples (Figure 2), using the four quantification methods. Since the 10 HPS samples were identical, the deviation of protein drifts from zero represented the analytical variability in the experiment. The mean of protein drifts, plotted as a solid line in the inserts of Figure 2, measured the strength of variation that affected all proteins similarly (i.e., the strength of systematic variation). The 95% confidence interval (CI) of protein drifts, plotted as a shaded band in the inserts of Figure 2, measured the strength of variation that affected different proteins differently (i.e., the strength of protein-specific variation). In comparison with the protein drifts for the raw MS data (Figure 2A), the protein drifts for EPN had a lower absolute mean but a comparable 95% CI (Figure 2B) while the protein drifts for SISQuan had a lower 95% CI but a comparable absolute mean (Figure 2C). Thus, EPN effectively controlled systematic variation and SISQuan effectively controlled protein-specific variation, illustrating the complementary nature of the two methods. The protein drifts for InteQuan had a lower absolute mean and a lower 95% CI (Figure 2D), illustrating that InteQuan suppressed both systematic and protein-specific variation.

### Improvement on precision of protein measurement

To assess the precision of InteQuan and SISQuan, CVs of the target proteins were evaluated from the 10 HPS samples (Table 2). InteQuan had better precision than SISQuan on all proteins except for ISLR. The median CV of all proteins was 9.3% using InteQuan versus 13.3% using SISQuan. InteQuan was statistically more precise than SISQuan (P = 5.2×10^-4^) and lowered protein CV by a median value of 4.9%. Using InteQuan, the highest CV was 16.8% (FRIL, 12 ng/ml). CVs of the remaining 15 target proteins were all below 15%, including 10 proteins with a CV below 10% and two proteins with a CV at or below 5%.

**Table 2:** **Coefficient of variation (CV) of protein abundance as evaluated using InteQuan and using SISQuan**

	Study I		Study II		Study III				Combined HPS dataset			
	CV (%)		CV (%)		CV (%)		Generalized CV (%)		CV (%)		Generalized CV (%)	
Protein	InteQuan	SISQuan	InteQuan	SISQuan	InteQuan	SISQuan	InteQuan	SISQuan	InteQuan	SISQuan	InteQuan	SISQuan
KIT	8.8	9.6	4.3	10.5	7.8	35.0	7.7	6.5	10.5	31.7	10.6	12.8
FRIL	16.8	25.2	5.3	11.8	7.3	33.6	7.3	12.0	15.9	25.4	16.7	18.8
COIA1	12.6	17.8	9.0	9.7	10.4	38.5	10.2	14.1	16.1	31.1	16.6	17.5
PRDX1	10.7	15.4	4.9	11.8	9.6	32.2	9.4	14.6	31.9	15.8	31.8	15.5
TENX	11.1	13.6	8.8	12.7	10.7	26.6	10.8	11.1	10.7	32.3	10.6	15.2
ENPL	13.1	18.9	11.2	8.6	11.3	34.9	11.3	13.6	11.7	32.9	11.8	12.6
GRP78	6.8	11.4	19.3	24.2	11.5	33.7	11.2	12.9	13.9	28.5	13.6	18.4
BGH3	5.0	12.3	5.7	9.9	12.4	42.3	12.4	13.8	9.8	33.7	9.6	14.9
ALDOA	6.6	13.6	9.1	17.5	15.1	35.4	14.9	19.9	11.0	35.4	11.2	14.9
GGH	6.9	7.1	9.0	13.7	13.9	38.9	13.7	16.5	11.0	31.7	10.5	16.6
CD14	4.1	8.0	4.6	12.0	4.6	35.3	4.7	6.8	7.9	30.5	7.8	11.9
LG3BP	8.8	13.0	5.9	10.0	5.6	31.1	5.6	7.5	8.6	30.5	8.5	13.9
TSP1	11.6	18.3	12.9	17.0	21.5	45.7	20.9	21.9	20.3	41.1	22.9	25.3
IBP3	5.7	11.6	6.3	13.5	13.5	41.5	14.0	15.5	19.8	26.7	20.5	21.6
TETN	9.9	17.8	9.9	12.4	25.5	52.3	26.9	29.7	33.1	47.6	33.3	37.7
ISLR	10.0	9.1	4.7	10.0	4.2	31.3	4.2	5.0	12.2	33.8	12.1	9.6
Median CV (%)	9.3	13.3	7.6	11.9	11.0	35.1	11.0	13.7	11.9	31.7	11.9	15.3
Median of CV reduction^*a*^ (%)	4.9		4.8		25.9		2.0		21.0		3.0	
Total proteins with lower CV	15	1	15	1	16	0	15	1	15	1	14	2
P value (paired sign test)	5.2 × 10^-4^		5.2 × 10^-4^		3.1 × 10^-5^		5.2 × 10^-4^		5.2 × 10^-4^		4.2 × 10^-3^	
Comments	CV of 10 HPS aliquots		Median CV of 15 clinical samples		Median CV of 6 samples				CV of 29 HPS aliquots			

^*a*^CV reduction was defined as CV using SISQuan minus CV using InteQuan.

### Improvement on panel performance in disease diagnosis

To illustrate the benefit of using InteQuan in clinical applications, a protein panel was constructed of all 16 target proteins and tested on the clinical samples in Study I using Monte Carlo cross validation (MCCV) [27]. Since the sample size was very small, the panel was not optimized for intended use, owing to concerns on both high false positive rate and high false negative rate. Using either InteQuan or SISQuan, the performance of the panel was summarized by the two receiver operating characteristic (ROC) curves in Additional file 4: Figure S2. The corresponding AUC was 0.573 (95% CI 0.569–0.576) using InteQuan or 0.528 (95% CI 0.524–0.532) using SISQuan, respectively. The improvement by InteQuan was 0.045 (95% CI 0.042–0.048, P < 0.0001). Thus, the panel had a significantly better performance using InteQuan than using SISQuan. More importantly, the ROC curve using InteQuan was consistently better than the ROC curve using SISQuan everywhere: See Additional file 4: Figure S2. This comparative analysis demonstrated that InteQuan improved the performance of the 16-protein panel in disease diagnosis, illustrating its value for biomarker research, despite the fact that the panel was not optimized for clinical application. A protein panel comprising a subset of the 16 target proteins was recently optimized and validated [23], using the InteQuan quantification method.

### Better control of analytical variability

To determine whether InteQuan can better control analytical variability during use of multiple depletion columns on clinical samples, 18 clinical samples in triplicate along with 12 aliquots of the HPS sample were analyzed in three experimental batches using three depletion columns in Study II (Figure 1C and Additional file 2: Table S1). The three aliquots of the clinical samples were processed either using different depletion columns or using the same column but at different positions in the depletion sequence, monitoring analytical variability due to column or position difference. Out of the 66 samples, an HPS sample and three clinical samples were lost during processing (Additional file 2: Table S1). A new lot of SIS peptide mixture was used in this study. The correction factors between the new and the old lots of SIS peptide mixture were determined from a migration experiment and are listed in Table 1. In this study we used this dataset to compare different quantification methods. More detailed variation analysis (using InteQuan only) will be presented elsewhere.

The median CVs of the target proteins were evaluated from the 15 clinical samples having three replicate measurements (Table 2). InteQuan demonstrated better precision than SISQuan on all proteins except for ENPL. The median CV of all proteins was 7.6% using InteQuan versus 11.9% using SISQuan. InteQuan was statistically more precise than SISQuan (P = 5.2×10^-4^) and lowered protein CV by a median value of 4.8%. Using InteQuan, the highest CV was 19.3% (GRP78, 100 ng/ml). CVs of the remaining 15 target proteins were all below 15%, including 13 proteins with a CV below 10% and four proteins with a CV below 5%.

**Figure 3:**
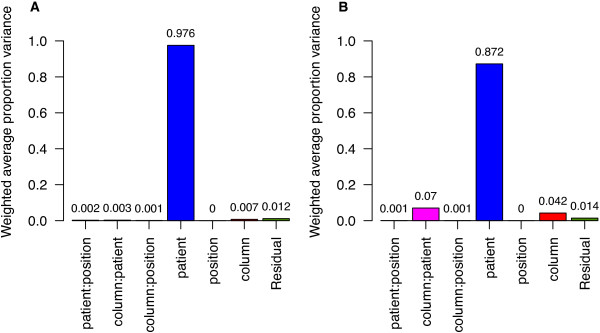
**Principal variance component analysis (PVCA) of protein abundance in the 15 clinical samples having three replicate measurements in Study II.** Protein abundance was evaluated using **(A)** InteQuan or **(B)** SISQuan.

To assess whether InteQuan can better control analytical variability without compromising its ability to reveal biological difference among the clinical samples, principal variance component analysis (PVCA) [28–30] was carried out to identify the major sources of variation in the experiment, including biological variation among individual patients (denoted as "patient"), analytical variation among depletion columns (denoted as "column"), and analytical variation among positions within a depletion sequence (denoted as "position") (Figure 3). For InteQuan, "patient" alone contributed 97.6% to the total variability while other sources jointly contributed a negligible fraction of 2.4%. For SISQuan, "patient" alone contributed 87.2% to the total variability while other sources jointly contributed 12.8%. Thus, InteQuan enhanced the ability of measuring biological difference among the clinical samples, in agreement with the previous observation that InteQuan improved the performance of the 16-protein panel in Study I. In other words, InteQuan improved the resolution of protein measurement in clinical samples.

### High tolerance against variation in total protein concentration

To demonstrate InteQuan’s ability to handle the variation in the total protein concentration, six samples were diluted into three concentrations (equivalent to the loading of 15, 20, or 30 μl of the original plasma samples: see Methods) and analyzed in duplicate using two depletion columns along with eight aliquots of the HPS sample in Study III (Figure 1C and Additional file 2: Table S1). No data was collected on one of the 44 samples owing to sample exhaustion. Manual review of experimental data identified two erratic measurements (Additional file 5: Figure S3A, B) that were eliminated from further analysis.

The median CVs of the target proteins were evaluated from the six samples using all valid measurements (Table 2). The median CV of all proteins was 11.0% using InteQuan and 35.1% using SISQuan. As a reference, the CV evaluated from the equivalent loading volumes (duplicates of 15, 20, and 30 μl) was 31.5%. While the median CV using SISQuan was higher than the CV of the loading volume, the median CV using InteQuan was much lower. Using InteQuan, all proteins had a median CV less than 20% except for TETN (25.5%) and TSP1 (21.5%), despite a two-fold difference in the total protein concentration.

### Usage of generalized CV for precision evaluation in study III

**Figure 4:**
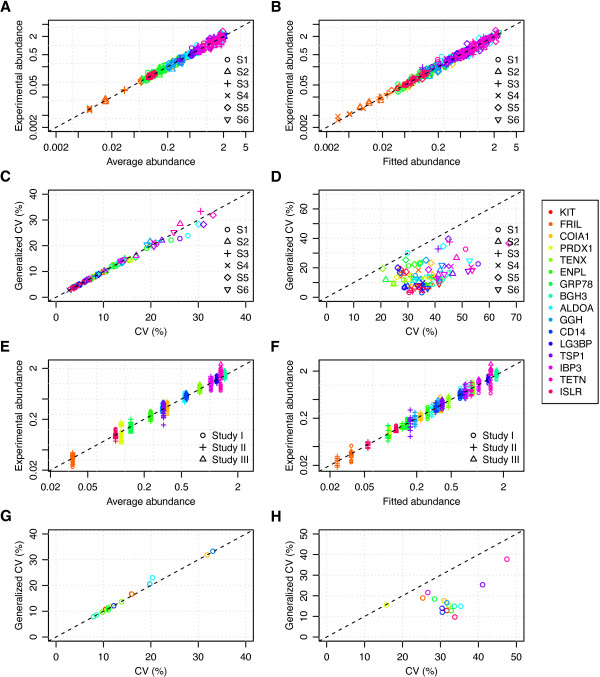
**Calculation of generalized coefficient of variation (CV). (A-D)** Results of all six clinical samples in Study III. (**E-H**) Results of the 29 HPS samples across all three studies. **(A, E)** Average InteQuan abundance versus experimental InteQuan abundance of individual proteins in individual samples. **(B, F)** Fitted SISQuan abundance versus experimental SISQuan abundance of individual proteins in individual samples. **(C, G)** The standard CV versus the generalized CV of InteQuan abundance. **(D, H)** The standard CV versus the generalized CV of SISQuan abundance.

The high CVs of the target proteins using SISQuan in Study III reflected the large difference in the total protein concentration (Additional file 5: Figure S3) rather than the precision of SISQuan. To compare the precision of InteQuan and SISQuan, a generalized method for CV calculation was developed. This method included two steps: First, the abundance of proteins in a sample was modeled either as linear functions of the loading volume (SISQuan) or as constants independent of the loading volume (InteQuan). Second, error propagation theory was applied to calculate the generalized CV as the standard deviation of differences between the modeled and the experimental abundances after logarithmic transformation. The modeled and the experimental abundances of all proteins in all samples collapsed nicely onto the respective diagonal line in Figure 4A and B, indicating that the method worked very well for both InteQuan and SISQuan. For SISQuan, it also demonstrated that proteins were measured within the respective linear dynamic range of the assays at all three concentrations. The generalized CVs and the standard CVs of InteQuan abundance were almost identical for all proteins in all samples (Figure 4C). On the contrary, the generalized CVs of SISQuan abundance were uniformly lower than the corresponding standard CVs (Figure 4D).

The median generalized CVs of the target proteins were evaluated from the six samples using all valid measurements (Table 2). InteQuan demonstrated better precision than SISQuan on all proteins except for KIT. The median generalized CV of all proteins was 11.0% using InteQuan versus 13.7% using SISQuan. InteQuan was statistically more precise than SISQuan (P = 5.2×10^-4^) and lowered protein generalized CV by a median value of 2.0%.

The generalized CV can be applied to analyze data from dilution experiments [2–4] within the linear dynamic range and to provide an assessment on precision over the whole concentration range. Ideally, generalized CV should be evaluated on data covering three or more concentrations to avoid over-fitting.

### Robustness of the depletion-MRM-MS platform

The three assessment studies lasted over six months, were carried out by different operators, encountered major instrument repairs, required implementation of a protocol change in sample loading volume, and used different reagent lots (Additional file 6: Table S3).

To assess the robustness of the depletion-MRM-MS platform, the CVs and the generalized CVs of the target proteins were computed from the 29 HPS samples across all three studies (Figure 4E**-**H and Table 2). Using InteQuan, 13 of the 16 target proteins had a CV less than 20%, 10 had a CV less than 15%, and three had a CV less than 10%. Only three proteins had a CV greater than 20%, including TSP1 with a CV of 20.3%, PRDX1 with a CV of 31.9%, and TETN with a CV of 33.1%.

We investigated possible causes for the high CVs of PRDX1 and TETN. On PRDX1, we noticed that, despite a lower loading volume per sample in Study II that was only two thirds of the loading volume in the other two studies, its SISQuan abundance was almost the same in all three studies. As a result, its InteQuan abundance was about 77% higher in Study II than in the other two studies, which led to the large CV value. The CV of its EPN abundance was only 16.7%. Thus, the large CV of its InteQuan abundance was likely due to issues on isotopic labeling rather than protein normalization. Possible causes for the inflated PRDX1 abundance in Study II include: (i) the correction factor for PRDX1 in Table 1 was incorrectly determined and/or (ii) the SIS peptide of QITVNDLPVGR of PRDX1 was partially cyclized [31] in Study II. On TETN, we noticed that the generalized CV of its SISQuan abundance was even higher at 37.7%. It turns out that TETN partially binds to the IgY14-Supermix resin column [25]. Possible causes for the large CV of TETN include: (i) the binding affinity varied between different depletion columns and/or (ii) the binding affinity was sensitive to the loading volume. In both cases InteQuan, as a quantification method itself, was not the cause for the high CV values.

Based on generalized CV, InteQuan had better precision than SISQuan on all proteins except for PRDX1 and ISLR. The median generalized CV of all proteins was 11.9% using InteQuan versus 15.3% using SISQuan. The generalized CV likely overestimated the precision of SISQuan since linear functions were used to fit SISQuan abundances at only two different protein concentrations, instead of the desirable three or more concentrations to avoid over-fitting. Nevertheless, InteQuan was statistically more precise than SISQuan (P = 4.2×10^-3^) and lowered protein generalized CV by a median value of 3.0%. Based on standard CV, the superiority of InteQuan to SISQuan was even more significant (P = 5.2×10^-4^).

### EPN as an alternative to InteQuan

To compare the precision of all four quantification methods (raw MS data, EPN, SISQuan, and InteQuan), CVs of protein abundance were evaluated from data of the 10 HPS samples in Study I (Table 3). Among the four methods, InteQuan was statistically more precise than SISQuan (P = 5.2×10^-4^), SISQuan was only marginally better than EPN (P = 0.80), and EPN was significantly better than the raw data (P = 3.1×10^-5^). Thus, the four quantification methods were ranked by their precision in descending order as InteQuan, SISQuan, EPN, and the raw MS data.

**Table 3:** **Coefficient of variation (CV) of protein abundance as evaluated on the 10 HPS samples in Study I**

Protein (HUMAN)	CV (%)				CV reduction (%)		
	InteQuan	SISQuan	EPN	Raw	SISQuan-InteQuan	EPN-SISQuan	Raw-EPN
KIT	8.8	9.6	15.0	24.1	0.8	5.4	9.2
FRIL	16.8	25.2	21.9	28.0	8.4	-3.3	6.1
COIA1	12.6	17.8	20.3	27.2	5.2	2.5	7.0
PRDX1	10.7	15.4	12.1	19.7	4.7	-3.3	7.6
TENX	11.1	13.6	9.6	20.2	2.5	-4.0	10.6
ENPL	13.1	18.9	6.9	19.1	5.8	-12.0	12.2
GRP78	6.8	11.4	16.7	22.5	4.5	5.3	5.8
BGH3	5.0	12.3	16.7	23.3	7.3	4.4	6.5
ALDOA	6.6	13.6	17.7	28.6	7.0	4.1	11.0
GGH	6.9	7.1	6.8	17.2	0.2	-0.3	10.3
CD14	4.1	8.0	21.1	31.8	3.8	13.1	10.7
LG3BP	8.8	13.0	8.8	19.4	4.2	-4.2	10.6
TSP1	11.6	18.3	19.6	22.5	6.7	1.3	2.8
IBP3	5.7	11.6	5.7	12.4	5.9	-5.9	6.6
TETN	9.9	17.8	19.6	21.7	7.9	1.8	2.1
ISLR	10.0	9.1	13.5	20.5	-0.9	4.4	7.0
**Median**	**9.3**	**13.3**	**15.9**	**22.1**	**4.9**	**1.6**	**7.3**
**P value (paired sign test)**					**5.2 × 10** ^**-04**^	**0.80**	**3.1 × 10** ^**-05**^

The above results suggested that the label-free EPN could be an attractive alternative to InteQuan, especially when it was too costly to obtain SIS peptides for hundreds to thousands of proteins of interest in early-stage biomarker discovery studies [7]. Using EPN, the median CV of all proteins was 15.9%. Three proteins had a CV just above 20%, including FRIL (12 ng/ml, 21.9%), CD14 (420 ng/ml, 21.1%), and COIA1 (35 ng/ml, 20.3%). CVs of the remaining 13 target proteins were all below 20%, including eight proteins with a CV at or below 15% and five proteins with a CV below 10%.

To further assess EPN, Pearson correlation coefficients of protein abundance as evaluated using different quantification methods were calculated on data of the 55 clinical samples in Study I (Table 4). The median Pearson correlation coefficient between InteQuan and EPN was 0.843. The lowest coefficient between them was 0.621 (CD14, P = 4.3×10^-7^). So the correlation between InteQuan and EPN was significant for all the target proteins.

**Table 4:** **Pearson correlation coefficient of protein abundance as evaluated on the 55 clinical samples in Study I**

Protein (HUMAN)	InteQuan vs. EPN	InteQuan vs. SISQuan	InteQuan vs. Raw	EPN vs. SISQuan	EPN vs. Raw	SISQuan vs. Raw
KIT	0.789	0.669	0.502	0.630	0.625	0.883
FRIL	0.963	0.919	0.862	0.933	0.898	0.971
COIA1	0.801	0.735	0.630	0.712	0.784	0.888
PRDX1	0.965	0.979	0.958	0.977	0.986	0.990
TENX	0.818	0.811	0.663	0.641	0.742	0.805
ENPL	0.936	0.883	0.838	0.781	0.831	0.893
GRP78	0.850	0.802	0.630	0.741	0.774	0.841
BGH3	0.740	0.679	0.589	0.685	0.760	0.882
ALDOA	0.954	0.958	0.943	0.902	0.927	0.977
GGH	0.837	0.804	0.749	0.621	0.844	0.792
CD14	0.621	0.498	0.234	0.704	0.727	0.782
LG3BP	0.900	0.910	0.826	0.807	0.865	0.913
TSP1	0.972	0.951	0.954	0.928	0.945	0.992
IBP3	0.918	0.816	0.749	0.756	0.784	0.872
TETN	0.775	0.779	0.702	0.680	0.745	0.905
ISLR	0.737	0.637	0.518	0.634	0.678	0.890
**Median**	**0.843**	**0.808**	**0.725**	**0.726**	**0.784**	**0.889**

All the 55 clinical samples in Study I had matching data from a previous label-free study. Major differences between the two studies were described in Methods. Pearson correlation coefficients of protein abundance were computed on data from the 55 clinical samples, using InteQuan on data from Study I and using EPN on data from the discovery study (Table 5). The median Pearson correlation coefficient between the two studies was 0.821. All proteins had a correlation coefficient above 0.5 except for TETN (0.418, P = 1.5×10^-3^). Despite major differences between the two studies, the correlation between EPN and InteQuan was significant for all the target proteins. Correlations between all feasible quantification methods on the two datasets are also listed in Table 5. Based on this evidence, it was justified to use EPN as an economical alternative to InteQuan in early-stage biomarker discovery studies.

**Table 5:** **Pearson correlation coefficient of protein abundance between Study I and a discovery study** ^***a***^

Protein (HUMAN)	InteQuan vs. EPN	InteQuan vs. Raw	EPN vs. EPN	EPN vs. Raw	SISQuan vs. EPN	SISQuan vs. Raw	Raw vs. EPN	Raw vs. Raw
KIT	0.711	0.536	0.560	0.404	0.270	0.434	0.196	0.290
FRIL	0.953	0.829	0.850	0.781	0.815	0.857	0.721	0.791
COIA1	0.770	0.610	0.715	0.605	0.679	0.735	0.626	0.646
PRDX1	0.978	0.971	0.946	0.943	0.950	0.975	0.937	0.956
TENX	0.831	0.690	0.800	0.711	0.607	0.683	0.551	0.615
ENPL	0.648	0.652	0.629	0.638	0.499	0.647	0.432	0.560
GRP78	0.649	0.618	0.620	0.656	0.444	0.624	0.367	0.597
BGH3	0.521	0.364	0.216	0.202	0.284	0.523	0.204	0.363
ALDOA	0.900	0.882	0.868	0.847	0.865	0.899	0.826	0.870
GGH	0.835	0.622	0.830	0.666	0.656	0.717	0.745	0.755
CD14	0.841	0.395	0.588	0.412	0.543	0.642	0.330	0.411
LG3BP	0.921	0.836	0.833	0.772	0.904	0.935	0.854	0.911
TSP1	0.909	0.802	0.876	0.750	0.918	0.902	0.918	0.884
IBP3	0.811	0.664	0.750	0.598	0.515	0.609	0.457	0.486
TETN	0.418	0.416	0.277	0.289	0.353	0.551	0.443	0.581
ISLR	0.783	0.651	0.700	0.637	0.554	0.712	0.500	0.624
**Median**	**0.821**	**0.652**	**0.733**	**0.647**	**0.581**	**0.698**	**0.526**	**0.620**

^*a*^Evaluated on the 55 common clinical samples between the two studies and labeled as method on data of Study I versus method on data of the discovery study.

## Conclusions

Three aspects of this study enhanced its relevance to development of blood-based laboratory-developed tests [32] using MRM-MS platforms. First, the target proteins were all potential lung cancer biomarkers [6]. Second, endogenous proteins in low ng/ml to low μg/ml plasma concentrations were quantified in both clinical plasma samples and in the standard HPS samples. Third, the longitudinal assessment on the robustness of the depletion-MRM-MS platform was performed in settings similar to actual laboratory operations for clinical testing. In contrast, medium- to high-abundant endogenous proteins, spike-in peptides or spike-in proteins were quantified on single plasma samples, in settings of academic research rather than clinical testing, and using different MRM-MS platforms in other studies [1–5]. Nevertheless, the precision obtained in this study was comparable to the precision reported in those studies. Furthermore, the precision of the whole depletion-MRM-MS platform was assessed in this study, not just the precision of MRM-MS platforms as in some studies.

The selection of proper endogenous normalizing proteins was crucial to the advantageous performance of InteQuan. According to error propagation theory, normalization by endogenous proteins has two opposite effects. On one hand, it reduces the overall variability in protein measurement by cancelling out systematic variation that similarly affects target and normalizing proteins. On the other hand, it increases the overall variability by transferring protein-specific and random variation of normalizing proteins to target proteins. Thus, normalization by endogenous proteins may not reduce the overall variability per se, as observed by others [19]. We applied the following three strategies to ensure the favorable outcome from the normalization process: First, we generated a large dataset to capture both technical variability on the platform and biological variability of the intended patient population. Owing to considerations of cost, a label-free approach was used to quantify proteins in the study [6]. Second, we selected the six normalizing proteins for their performance in reducing column drift and technical CV of other proteins. In other words, the proteins were specifically selected to fulfill the role of normalizers. Third, we used the six normalizing proteins as a panel that was more stable compared to individual proteins. In addition, although plasma concentration was not used as a selection criterion, the wide concentration range (three orders of magnitude) of the six normalizing proteins was likely beneficial [20]. Similar strategies should be used for selecting endogenous normalizing proteins on other MS platforms and/or for other proteomics projects. In our case, the six normalizing proteins were selected from a pool of 371 protein candidates based on a set of label-free depletion-MRM-MS data [6]. It is possible that other proteins outside the pool may be better normalizers and/or that some of the six proteins are not good normalizers on other MS platforms.

InteQuan measured the abundance of the target proteins relative to the abundance of the endogenous normalizing proteins, which explains its high tolerance against variation in the total protein concentration. When testing actual clinical samples, pre-analytical variability (due to differences [10] in patient posture, diurnal cycle, sample collection, and/or sample handling, etc.) and analytical variability (due to differences in sample loading volume, instrument performance, and/or operator, etc.) are hard to avoid and all contribute to the overall variability of the assay. Thus, a high tolerance against such variation is a desirable feature that will increase the reproducibility of clinical tests. Many high-impact multiplex clinical tests on transcriptomic platforms used similar strategies of quantifying genes of interest relative to a set of reference genes in clinical samples [33, 34].

In summary, we have developed InteQuan as a quantification method for MS-based quantitative proteomics and demonstrated its superiority to SISQuan in three independent studies and on the combined HPS dataset. The method is robust, simple to implement, capable of reducing pre-analytical and analytical variability, and able to improve the measurement of biological differences. All these features make the method an ideal technique for MS-based quantitative proteomics, especially for applications in biomarker research and in routine clinical testing.

## Methods

### Clinical samples

Archival K2-EDTA plasma samples were obtained from subjects that provided informed consent and with approval by either the Ethics Review Board at Institut Universitaire de Cardiologie et de Pneumologie de Quebec or the Institutional Review Boards at New York University and University of Pennsylvania. All samples were collected prior to surgery or from patients without surgery. Disease status of patients was histopathologically confirmed. All cancer patients were at Stage I or II. Clinical data associated with subjects were handled in accordance with the guidance established by the Health Insurance Portability and Accountability Act of 1996 to ensure subject privacy.

### Selection of endogenous normalizing proteins

In our previous discovery study [6], 72 cancer and 71 benign samples were analyzed in five experimental batches along with 15 aliquots of a pooled HPS sample that was purchased from Bioreclamation (Hicksville, NY). The HPS samples were embedded among clinical samples and analyzed repeatedly to monitor analytical variability in the experiment. The clinical samples were used to represent biological variability and possible pre-analytical variability.

Endogenous normalizing proteins were selected from proteins whose strongest transitions were detected in all samples. Each protein candidate was used to normalize the abundance of other proteins and evaluated based on the following criteria: (A) Its rank, as a normalizer, in reducing median technical CV of other proteins; (B) its rank in compensating median column drift [6], that is a technical variation associated with depletion; (C) its own median technical CV on HPS samples; and (D) its own median biological CV on clinical samples. In the end, six endogenous normalizing proteins were selected: See Figure S5 and Table S11 of reference [6]. Owing to considerations of cost, the selection of endogenous normalizing proteins was performed in a label-free approach.

### Immunoaffinity chromatography

Experimental protocols for sample preparation were adapted and modified from a recent study [6]. Immunoaffinity columns containing a 2:1 ratio of IgY14 and SuperMix resins were purchased from Sigma Aldrich (St. Louis). Each column was conditioned with 0.15 M (NH_4_)HCO_3_ at 0.5 ml/min for 45 min. Prior to immunoaffinity separation of each sample batch, column performance was assessed with replicate injections of aliquots of the HPS sample.

To isolate low abundance proteins, 45, 50, or 60 μl of plasma were diluted in 0.15 M (NH_4_)HCO_3_ to a final volume of 135, 150, or 180 μl, respectively, and filtered using a 0.45 μm AcroPrep 96-well filter plate (Pall Life Sciences). Immunoaffinity separation was conducted on a IgY14-SuperMix column connected to an high performance liquid chromatography (HPLC) system (Agilent 1260 Infinity Bioinert Quaternary liquid chromatography (LC)) using 3 buffers (loading/washing: 0.15 M (NH_4_)HCO_3_; stripping/elution: 0.1 M glycine, pH 2.5; and neutralization: 0.01 M Tris-HCl and 0.15 M NaCl, pH 7.4) with a cycle comprised of load, wash, elute, neutralization, and re-equilibration lasting 36 min. The total plasma volume loaded onto the depletion column was 15, 20, or 30 μl, respectively. The unbound and bound fractions were monitored at 280 nm and were baseline resolved after separation. Unbound fractions (containing the low abundance proteins) were collected for downstream processing and analysis and lyophilized prior to enzymatic digestion. Every 24 samples were grouped as an experimental batch and were processed sequentially in a throughput of one batch per day.

### Enzymatic digestion

Lyophilized fractions containing low abundance proteins were digested with trypsin after being reconstituted under mild denaturing conditions in 200 μl of 1:1 0.1 M (NH_4_)HCO_3_ /trifluoroethanol (TFE) (v/v) and then allowed to incubate on an orbital shaker for 30 min at room temperature (RT). Samples were diluted in 800 μl of 0.1 M (NH_4_)HCO_3_ and digested with 0.4 μg trypsin (Princeton Separations) per sample for 16+/-2 hours at 37°C. Following digestion samples were stored at -70°C for 2 hours and then lyophilized. Samples within each study were digested in parallel.

### Stable isotope-labeled standard peptides

A total of 26 SIS peptides were purchased from New England Peptide (Gardner, MA), including one SIS peptide for each of the six normalizing proteins and the 18 target proteins in Table 1. SIS peptides of two additional proteins (S10A6 and PROF1) were included as potential biomarkers earlier on but were later eliminated. Each SIS peptide was purified to 95% or greater as determined by reversed phase HPLC; mass determination for each peptide was confirmed to be within 0.1% of the calculated mass by matrix-assisted laser desorption/ionization (MALDI)-time of flight (TOF) MS. The concentration of the stock solution for each peptide was determined by amino acid analysis. The SIS peptide mixture was produced per specified formulation in 10% acetonitrile, 0.1% formic acid final concentration with 100 fmol/μL BSA digest added for stability. Concentrations of individual SIS peptides were tailored so that their MRM-MS signal intensities were comparable to those of the corresponding endogenous peptides. The mixture was aliquoted into individual 300 μL single use microfuge tubes and stored at -80°C. Aliquots of the SIS peptide mixture were thawed on wet ice, mixed briefly and spiked into peptide samples after enzymatic digestion and lyophilization and during solubilization just prior to solid-phase extraction. Two different preparations (lots) of the SIS peptide mixture were prepared and used in this study. The stability of SIS peptides was monitored based on their MRM signal intensities. No evidence for the instability of SIS peptides was observed over a period of 20 months (data not shown).

### Solid-phase extraction

Aliquots of the SIS peptide mixture were spiked into the lyophilized peptide samples, followed by reconstitution in 350 μl of 0.01 M (NH_4_)HCO_3_, incubation on an orbital shaker for 15 min at RT, reduction using 30 μl of 0.05 M TCEP, incubation for 1 hour at RT, and dilution in 375 μl of 90% water/10% acetonitrile/0.2% trifluoroacetic acid. The solid phase extraction plate (Empore C18, 3 M Bioanalytical Technologies) was conditioned according to the manufacturer’s protocol, and after sample loading were washed in 500 μl of 95% water/5% acetonitrile/0.1% trifluroacetic acid and eluted by 200 μl of 52% water/48% acetonitrile/0.1% trifluoroacetic acid into a collection plate. The eluate was split into 2 equal aliquots and was taken to dryness in a vacuum concentrator. One aliquot was used immediately for mass spectrometry, while the other was stored at -80°C. Samples were reconstituted in 12 μl of 90% water/10% acetonitrile/0.2% formic acid just prior to LC-MRM-MS analysis. Samples within each study were processed in parallel in this step.

### Optimization of MRM assays

MRM assays of endogenous peptides of the target and normalizing proteins were developed previously on a 5500 QTrap® reversed-phase LC-MRM-MS platform (AB Sciex) [6]. The specificity of the assays was verified with a FDR of 3.70% or lower. These assays, along with MRM assays of the corresponding SIS peptides, were transferred to and optimized on a 6490 Triple Quadrupole LC-MRM-MS platform (Agilent) based on the highly purified synthetic SIS peptides [8, 35, 36]. The optimal assays were further tested on processed HPS samples to check for signal intensity and possible interference. Unless specified, the signal of the assays was well above noise and within the respective linear dynamic range. In addition to the low FDRs of the original assays, the specificity of the transitions to the corresponding proteins was further verified from the co-elution of endogenous and SIS peptides and from the consistency between the peptides on intensities of different transitions. Seventeen additional proteins were analyzed for exploratory purposes without optimizing their transitions or spiking in the corresponding SIS peptides. The 17 extra proteins were not analyzed in this study. A total of 302 transitions from 38 proteins were measured in this study.

### MRM-MS analysis

Peptide samples were separated using a capillary reversed-phase LC column (Agilent Poroshell 120 EC-C18; 2.1 mm ×100 mm, particle size 2.7 μm) and an Agilent 1290 Infinity HPLC system. The mobile phases were (A) 0.1% formic acid in water and (B) 0.1% formic acid in acetonitrile. The samples were injected (8 μl) and separated using a linear gradient (98% A to 70% A) at 0.4 mL/minute for 21.7 min. Peptides were eluted directly into the electrospray source of the mass spectrometer (6490 Triple Quadrupole, Agilent) operating in scheduled MRM positive-ion mode (Q1 resolution: wide; Q3 resolution: unit; detection window variable: 124 to 240 seconds; cycle time: 1.0 seconds). Peak areas of transitions were integrated by MassHunter (Agilent) and manually curated to ensure quality. Samples within each experiment were analyzed sequentially.

### Four quantification methods

In this study the abundance of a protein was evaluated based on the MRM signal intensity of the strongest transition of the protein and no distinctions between protein abundance, peptide abundance and transition abundance were made. Without losing generality, the four quantification methods were described in terms of peptide quantification as follows.

#### Raw MS data

In this label-free quantification approach, the abundance of peptide *p* in sample *s* was measured by its raw peak area (*A*_*p*,*s*_) without normalization.

#### Endogenous protein normalization (EPN)

In this label-free quantification approach, the abundance of peptide *p* in sample *s* was measured by its normalized peak area , where  was a sample-dependent normalization factor and was calculated from the peak areas of a predetermined set of *N* = 6 endogenous, normalizing peptides in the sample. More specifically,
1

where *A*_*n*,*s*_ was the peak area of peptide normalizer *n* (with *n* = 1, …, *N*) in the sample and *Ă*_*n*_ was a scaling constant for the normalizer that ensured values of {*A*_*n*,*s*_/*Ă*_*n*_} among all normalizers to be the same on average. The scaling constants {*Ă*_*n*_} were determined as the median values (over all clinical samples) of {*A*_*n*,*s*_} in an independent study of 120 samples [23].

#### Quantification using SIS peptides (SISQuan)

In this labeled quantification approach, the abundance of peptide *p* in sample *s* was measured by the response ratio between the endogenous peptide to the corresponding SIS peptide, that is *R*_*p*,*s*_ = *A*_*p*,*s*_/*Ă*_*p*,*s*_ where *Ă*_*p*,*s*_ was the peak area of the SIS peptide.

#### Integrated quantification (InteQuan)

In this labeled quantification approach, the abundance of peptide *p* in sample *s* was measured by its normalized response ratio , where  was a sample-dependent normalization factor and was calculated from the response ratios of the *N* peptide normalizers in the sample. More specifically,
2

where *R*_*n*,*s*_ was the response ratio of peptide normalizer *n* in the sample and  was a scaling constant for the normalizer that ensured values of  among all normalizers to be same on average. Similar to {*Ă*_*n*_}, the scaling constants  were determined as the median values (over all clinical samples) of {*R*_*n*,*s*_} in the same study of 120 samples [23].

### Migration to new lot of SIS peptides

Six aliquots of the HPS sample (30 μl per aliquot) were processed and pooled together after digestion. The pooled sample was split into two identical aliquots. Two lots of SIS peptide mixtures (old and new) were each spiked into one of the two aliquots of HPS. The two aliquots of SIS peptide/HPS mixture were then each further split into three equal aliquots and lyophilized. The SIS peptide/HPS mixtures were reconstituted, desalted, lyophilized, and stored. The SIS peptide/HPS samples were then solubilized and analyzed by MRM-MS. A correction factor was calculated for each peptide as , where  ( ) was the median response ratio of peptide *p* as evaluated using the old (new) lot of SIS peptides. In Study II, the abundance ratio *R*'_*p*,*s*_ of peptide *p* in sample *s* as measured using the new lot was multiplied by the correction factor *F*_*p*_, that is *R*_*p*,*s*_ = *R* ' _*p*,*s*_ * *F*_*p*_. This correction was applied to both the target and the normalizing peptides. Afterwards, the evaluation of protein abundance using InteQuan and using SISQuan were both based on the corrected abundance ratios {*R*_*p*,*s*_}.

### Intensity drift

The intensity drift of peptide *p* in sample *s* was defined as
3

where *I*_*p*,*s*_ was the abundance of the peptide in the sample and *Ĭ*_*p*_ was the corresponding median value in all technical replica. The intensity drift *D*_*p*,*s*_ evaluated how far the abundance of the peptide in the sample deviated from the overall median abundance of the peptide. The median value of *D*_*p*,*s*_ was zero by definition for all peptides.

### Monte Carlo cross validation

Monte Carlo cross validation (MCCV) [27] was performed as follows: First, all clinical samples in Study I were randomly assigned to a training group (including 24 benign and 24 cancer samples) or a test group (including 4 benign and 3 cancer samples). Second, two logistic regression models were developed to fit the disease status of the training samples, using either the InteQuan abundances or the SISQuan abundances of all 16 proteins in Table 2 as predictors. The first two steps were repeated if any one of the two models failed to converge. Third, the models were used to calculate scores of the test samples, evaluating their likelihood of being a cancer sample, based on protein InteQuan or SISQuan abundances, respectively. Fourth, the test samples were ranked by their scores from the InteQuan model or the SISQuan model, respectively. Fifth, the first four steps were repeated 10,000 times with different sample permutations. The ranking and the corresponding disease status of the test samples in all permutations were assembled under either InteQuan or SISQuan, respectively. Finally, comparison of ROC curves was carried out to compare the MCCV performance of the 16-protein panel using InteQuan with the corresponding performance using SISQuan. Due to small sample size, covariates in both the training samples and the test samples were unavoidable and difficult to adjust, which made it not meaningful to direct compare scores of the test samples between different permutations [37]. Thus, the ranking instead of the score was combined for the ROC analysis, which effectively standardized the scores between different permutations [38]. The ROC comparison analysis was performed by MedCalc (Ostend, Belgium), selecting "DeLong et al." [39] and "Binomial exact Confidence Interval for the AUC" as options.

### Calculation of generalized CV

The method consisted of two steps:

In the first step, protein InteQuan abundances in a sample were modeled as constants independent of the loading volume. Thus, the expected InteQuan abundances were assigned to the corresponding average values, that is
4

Here  was the InteQuan abundance of protein *p* in sample *s* at the loading volume *v*_*i*_ = 15, 20, *or* 30 and *N*_*p*,*s*_ was the number of repeat measurements of the protein on the sample regardless of the loading volumes, that is *N*_*p*,*s*_ = 5 *or* 6 in Study III and *N*_*p*,*s*_ = 29 for the combined HPS dataset.

On the contrary, protein SISQuan abundances in a sample were modeled as linear functions of the loading volume. More specifically, the expected SISQuan abundances were fitted as linear functions of the loading volume such that
5

Here *a*_*p*,*s*_ was proportional to the concentration of the protein in the sample and *b*_*p*_ was common to all samples. Parameters {*a*_*p,s*_} and *b*_*p*_ were evaluated from repeat measurements of the protein in all samples using maximum likelihood estimation [40]. Ideally one should have at least three loading volumes to avoid over-fitting.

In the second step, error propagation theory was applied to evaluate the generalized CV. According to the theory, the CV of a quantity equals to the standard deviation of the same quantity after logarithmic transformation, that is  where *σ*(*x*) represents the standard deviation of *x* and ln(*x*) is the natural logarithmic function. Thus, the generalized CV of protein abundance was evaluated from differences between the expected and the experimental values after logarithmic transformation. More specifically, the generalized CV of InteQuan abundance was evaluated as
6

And the generalized CV of SISQuan abundance was evaluated as
7

Here *K* was the number of different samples used in the study and was needed to account for the fitting of *b*_*p*_. Thus, *K* = 6 in Study III and *K* = 1 for the combined HPS dataset.

### Differences between Study I and a previous study

All clinical samples in Study I have been previously processed and analyzed by a contract research organization (CRO; Caprion, Montreal). Similar protocols were used in immunoaffinity depletion, protein digestion and desalting [6]. Major differences between the two studies included: 1) Laboratory: Study I was carried out in-house but the discovery study was done by the CRO. 2) Depletion: The depletion column was ordered directly from vendor in Study I but packed by the CRO with a different lot of IgY14-Supermix resin beads in the discovery study. 3) Quantification: SIS peptides were used for quantification in Study I but not in the discovery study. 4) MS platform: Peptides were analyzed by an Agilent 6490 Triple Quadrupole LC/MS System in Study I but by an AB SCIEX QTrap® 5500 LC/MS system in the discovery study. 5) Monitored transitions: 302 transitions of 38 proteins were monitored in Study I. In comparison, 1550 transitions of 344 proteins were monitored in the discovery study.

### Data analysis

Data analysis was performed using the R statistical environment. Code for PVCA [28–30] was adapted from: [http://www.niehs.nih.gov/research/resources/software/biostatistics/pvca/], setting the threshold to capture at least 90% of variance and a minimum of two principal components. The *p* value for comparing different quantification methods was based on the most-applicable, non-parametric paired sign test, assuming that measurements were independent and from a continuous population. The *p* value was evaluated using the function "SIGN.test" in the "BSDA" library. Functions "glm" and "predict" were used to train and test logistic regression models. Function "lm.fit" in the "stats" library was used to fit the linear relationship between the SISQuan abundances and the sample loading volumes.

### Data availability

Raw MS data in mzML format and the full list of MRM assays can be downloaded from SRMAtlas (http://www.peptideatlas.org/PASS/PASS00390).

## Electronic supplementary material

Additional file 1: Figure S1: Chromatograms of transitions of both endogenous and SIS peptides of individual proteins. (PDF 114 KB)

Additional file 2: Table S1: Sample layout in the three assessment studies. (PDF 50 KB)

Additional file 3: Table S2: Clinical information of patients in Study I. (PDF 49 KB)

Additional file 4: Figure S2: Receiver operating characteristic curves of the panel of all 16 target proteins, evaluated with Monte Carlo cross validation (MCCV) on clinical samples in Study I. (PDF 62 KB)

Additional file 5: Figure S3: Examples of SISQuan abundance versus loading volume in Study III. (PDF 52 KB)

Additional file 6: Table S3: Summary of main experimental differences among the three assessment studies and major instrument services. (PDF 42 KB)

## Abbreviations

AUC: Area under the curve
CI: Confidence interval
CRO: Contract research organization
CV: Coefficient of variation
Depletion-MRM-MS: Immunoaffinity-based protein depletion coupled with MRM-MS
EPN: Endogenous protein normalization
FDR: False discovery rate
HPLC: High performance liquid chromatography
HPS: Human plasma standard
InteQuan: Integrated quantification
LC: Liquid chromatography
MCCV: Monte Carlo cross validation
MRM: Multiple reaction monitoring
MS: Mass spectrometry
ROC: Receiver operating characteristic
RT: Room temperature
SIL: Stable isotope labeling
SIS: Stable isotope-labeled internal standard
SISQuan: Quantification using stable isotope-labeled internal standard peptides
PVCA: Principal variance component analysis.
