# Supplementary material for: An integrated quantification method to increase the precision, robustness, and resolution of protein measurement in human plasma samples
Source: Clin Proteomics. 2015 Jan 29;12(1):3. doi: 10.1186/1559-0275-12-3 (PMC4363461; doi:10.1186/1559-0275-12-3)
Supplement: Supplementary file 1 — Additional file 1: Figure S1: Chromatograms of transitions of both endogenous and SIS peptides of individual proteins. (PDF 114 KB) [file 12014_2014_91_MOESM1_ESM.pdf]

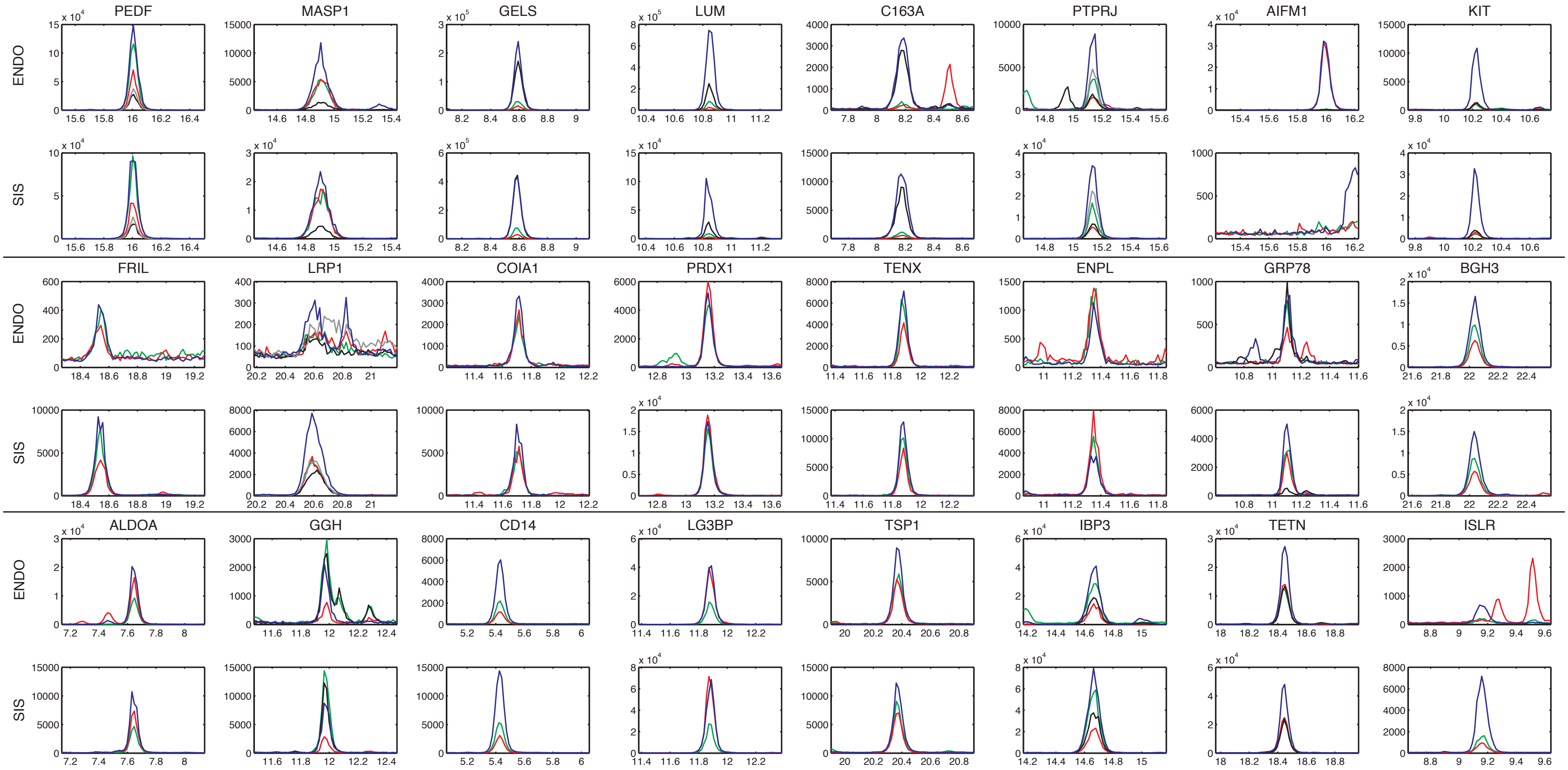


**Figure S1 Chromatograms of transitions of both endogenous and SIS peptides of individual proteins.** Proteins were plotted in the same order as in Table 1. Transitions of endogenous peptides were plotted in the first, third and fifth rows. Transitions of the corresponding SIS peptides were plotted in the second, fourth and sixth rows. Transitions used for protein quantification were plotted in blue.
