# Supplementary material for: An integrated quantification method to increase the precision, robustness, and resolution of protein measurement in human plasma samples
Source: Clin Proteomics. 2015 Jan 29;12(1):3. doi: 10.1186/1559-0275-12-3 (PMC4363461; doi:10.1186/1559-0275-12-3)
Supplement: Supplementary file 2 — Additional file 2: Table S1: Sample layout in the three assessment studies. (PDF 50 KB) [file 12014_2014_91_MOESM2_ESM.pdf]

Table S1 Sample layout in the three assessment studies[a,b]

Position	Study I			Study II			Study III[c]	
	Batch			Batch			Batch	
	1	2	3	1	2	3	1	2
1	HPS	HPS	HPS	HPS	HPS	HPS	HPS	HPS
2	S1	S21	S41	S1	S13	S16	S1-L1	S1-L0
3	S2	S22	S42	S2	S2	S2	S1-L0	S1-L2
4	S3	S23	S43	S3	S14	S17	S1-L2	S1-L1
5	S4	S24	S44	S4	S4	S4	S2-L0	S2-L1
6	S5	S25	S45	S5	S15	S18	S2-L2	S2-L0
7	S6	S26	S46	S6	S6	S6	S2-L1	S2-L2
8	HPS	HPS	HPS	HPS	HPS	HPS	HPS	HPS
9	S7	S27	S47	S1	S13	S16	S3-L2	S3-L0
10	S8	S28	S48	S7	S7	S7	S3-L1	S3-L2
11	S9	S29	S49	S3	S14	S17	S3-L0	S3-L1
12	S10	S30	S50	S8	S8	S8	S4-L1	S4-L2
13	S11	S31	S51	S5	S15	S18	S4-L0	S4-L1
14	S12	S32	S52	S9	S9	S9	S4-L2	S4-L0
15	S13	S33	S53	HPS	HPS	HPS	HPS	HPS
16	S14	S34	S54	S1	S13	S16	S5-L1	S5-L0
17	HPS	HPS	HPS	S10	S10	S10	S5-L2	S5-L1
18	S15	S35	S55	S3	S14	S17	S5-L0	S5-L2
19	S16	S36	S56	S11	S11	S11	S6-L0	S6-L1
20	S17	S37	S57	S5	S15	S18	S6-L1	S6-L2
21	S18	S38	S58	S12	S12	S12	S6-L2	S6-L0
22	S19	S39	S59	HPS	HPS	HPS	HPS	HPS
23	S20	S40	S60	NC	NC	NC	NC	NC
24	HPS	HPS	HPS	NC	NC	NC	NC	NC

[a]HPS, human plasma standard. NC, negative control (0.15 M $(NH_4)HCO_3$).

[b]Samples with no data were highlighted in box and were lost due to 1) unexpected pump stoppage and restarting during depletion (the first HPS in Study I), 2) sample exhaustion (the sample in Study III) or 3) sample contamination during solid-phase extraction (all other samples).

[c]The loading volume for samples labeled as L0, L1, and L2 was 30, 20 or 15 μl, respectively.
