# Supplementary material for: An integrated quantification method to increase the precision, robustness, and resolution of protein measurement in human plasma samples
Source: Clin Proteomics. 2015 Jan 29;12(1):3. doi: 10.1186/1559-0275-12-3 (PMC4363461; doi:10.1186/1559-0275-12-3)
Supplement: Supplementary file 3 — Additional file 3: Table S2: Clinical information of patients in Study I. (PDF 49 KB) [file 12014_2014_91_MOESM3_ESM.pdf]

Table S2 Clinical information of patients in Study I

Subject	Disease Status	Cancer Stage	Histopathology of Cancer	Gender	Age	Nodule Size (mm)	Smoking Status	Pack-Year
S1	Benign			Male	69	33	Past	98
S2	Cancer	I	ADENOCARCINOMA w/MIXED	Male	71	6	Past	25
S3	Cancer	I	SQUAMOUS	Male	53	45	Past	114
S4	Benign			Male	56	26	Past	10
S5	Benign			Female	63	35	Never	0
S6	Cancer	I	ADENOCARCINOMA	Female	64	39	Past	7.5
S7	Benign			Male	67	47.5	Current	55
S8	Cancer	II	SQUAMOUS	Male	72	43	Past	48
S9	Benign			Male	58	25	Current	40
S10	Cancer	I	ADENOCARCINOMA	Male	57	30	Past	40
S11	Cancer	I	ADENOCARCINOMA	Male	61	36	Past	20
S12	Benign			Male	55	22	Past	15
S13	Benign			Female	52	43	Never	0
S14	Benign			Female	46	40	Current	30
S15	Benign			Male	52	38	Past	9
S16	Cancer	II	MIXED	Male	48	40	Current	35
S17	Benign			Male	64	32	Past	12
S18	Cancer	II	SQUAMOUS	Male	62	24	Current	92
S19	Cancer	I	SQUAMOUS	Male	73	32	Past	130
S20	Benign			Male	74	22	Never	0
S21	Cancer	II	ADENOCARCINOMA	Female	67	40	Past	40
S22	Benign			Female	66	40	Never	0
S23	Cancer	I	ADENOCARCINOMA	Male	60	12	Past	17.5
S24	Benign			Male	62	40	Past	NA
S25	Benign			Female	76	40	Never	0
S26	Cancer	II	ADENOCARCINOMA	Female	75	44	Past	58
S27	Benign			Female	62	23	Past	NA
S28	Cancer	I	ADENOCARCINOMA	Female	58	22	Current	19
S29	Benign			Male	43	56	Current	30
S30	Cancer	II	SQUAMOUS	Male	51	58	Past	95
S31	Benign			Male	77	26	NA	45.6
S32	Cancer	I	ADENOCARCINOMA	Male	75	9	Past	91.5
S33	Cancer	II	ADENOCARCINOMA	Male	79	50	Past	12.5
S34	Benign			Male	78	48	Past	NA
S35	Cancer	II	NA	Female	56	24	Past	36
S36	Benign			Female	59	24	Past	47.5
S37	Benign			Female	64	26	Never	0
S38	Cancer	I	ADENOCARCINOMA	Female	64	33	Never	0
S39	Benign			Female	53	54	Past	30

Table S2 Clinical information of patients in Study I

Subject	Disease Status	Cancer Stage	Histopathology of Cancer	Gender	Age	Nodule Size (mm)	Smoking Status	Pack-Year
S40	Cancer	II	ADENOCARCINOMA	Female	72	32	Current	50
S41	Benign			Female	61	35	Past	25
S42	Cancer	II	ADENOCARCINOMA w/MIXED	Female	60	25	Current	90
S43	Benign			Female	38	22	Never	0
S44	Cancer	II	ADENOCARCINOMA	Female	75	45	Current	12.5
S45	Benign			Male	65	24	Past	30
S46	Cancer	I	ADENOCARCINOMA	Male	64	50	Past	NA
S47	Cancer	II	SQUAMOUS	Female	47	55	Never	0
S48	Benign			Female	52	24	Past	15
S49	Cancer	IIA	SQUAMOUS	Male	82	34	Past	30
S50	Benign			Male	84	32	Past	30
S51	Cancer	II	SQUAMOUS	Female	63	47	Past	15
S52	Benign			Male	44	45	Past	30
S53	Benign			Male	41	31	Past	20
S54	Cancer	II	ADENOCARCINOMA	Male	51	35	Past	30
S55	Cancer	II	SQUAMOUS	Female	67	52	Current	NA
S56	Benign			Female	65	42	Never	0
S57	Benign			Female	54	5	Past	20
S58	Cancer	II	ADENOCARCINOMA w/MIXED	Female	67	44	Past	30
S59	Benign			Female	60	41	Current	40
S60	Cancer	II	ADENOCARCINOMA	Female	61	27	Past	40
