# Supplementary material for: An integrated quantification method to increase the precision, robustness, and resolution of protein measurement in human plasma samples
Source: Clin Proteomics. 2015 Jan 29;12(1):3. doi: 10.1186/1559-0275-12-3 (PMC4363461; doi:10.1186/1559-0275-12-3)
Supplement: Supplementary file 4 — Additional file 4: Figure S2: Receiver operating characteristic curves of the panel of all 16 target proteins, evaluated with Monte Carlo cross validation (MCCV) on clinical samples in Study I. (PDF 62 KB) [file 12014_2014_91_MOESM4_ESM.pdf]

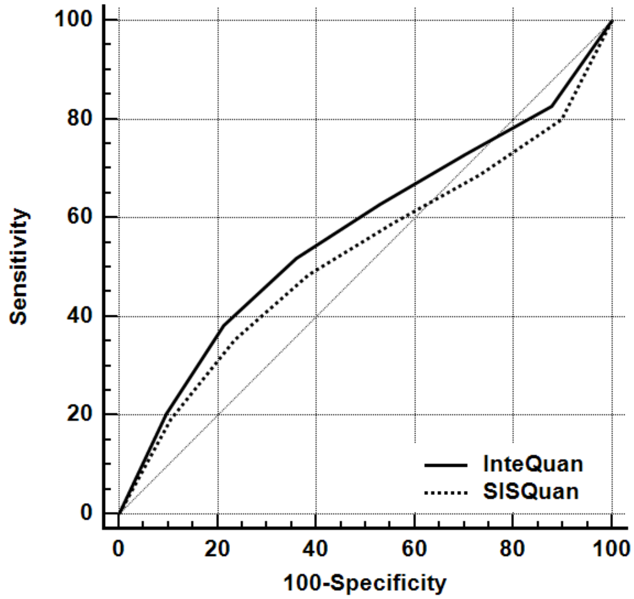

Figure S2 Receiver operating characteristic curves of the panel of all 16 target proteins, evaluated with Monte Carlo cross validation (MCCV) on clinical samples in Study I. Proteins were quantified using InteQuan (solid line) or SISQuan (dotted line).
