# Supplementary material for: An integrated quantification method to increase the precision, robustness, and resolution of protein measurement in human plasma samples
Source: Clin Proteomics. 2015 Jan 29;12(1):3. doi: 10.1186/1559-0275-12-3 (PMC4363461; doi:10.1186/1559-0275-12-3)
Supplement: Supplementary file 5 — Additional file 5: Figure S3: Examples of SISQuan abundance versus loading volume in Study III. (PDF 52 KB) [file 12014_2014_91_MOESM5_ESM.pdf]

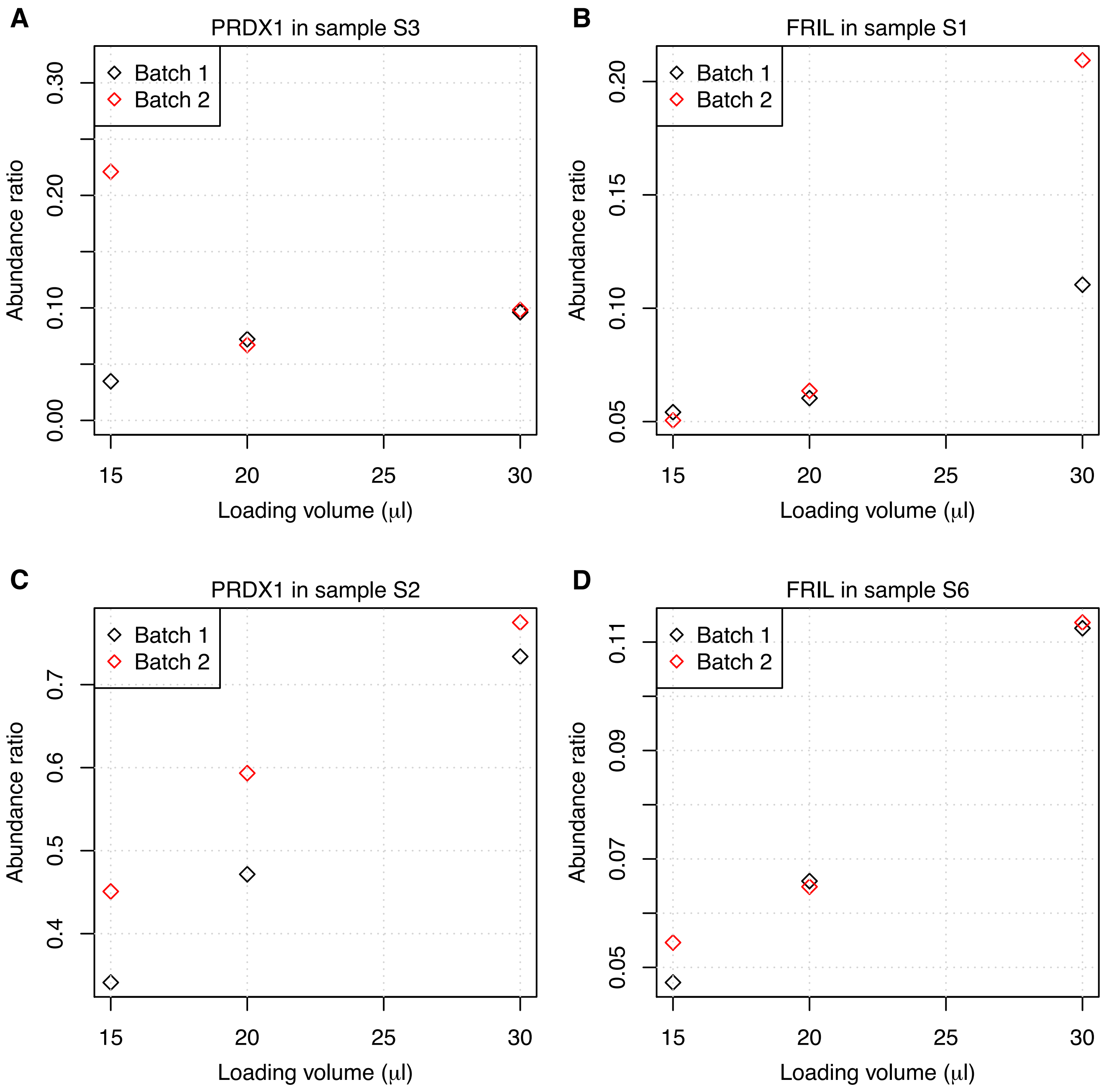

Figure S3 Examples of SISQuan abundance versus loading volume in Study III. The top row shows two outlier measurements while the bottom row shows the corresponding linear behavior of the same proteins. (**A**) PRDX1 in sample S3. The outlier measurement (Batch 2 with the loading volume of 15 μl) was removed from analysis. (**B**) FRIL in sample S1. The outlier measurement (Batch 2 with the loading volume of 30 μl) was removed from analysis. (**C**) PRDX1 in sample S2. (**D**) FRIL in sample S6.
