# Supplementary material for: An integrated quantification method to increase the precision, robustness, and resolution of protein measurement in human plasma samples
Source: Clin Proteomics. 2015 Jan 29;12(1):3. doi: 10.1186/1559-0275-12-3 (PMC4363461; doi:10.1186/1559-0275-12-3)
Supplement: Supplementary file 6 — Additional file 6: Table S3: Summary of main experimental differences among the three assessment studies and major instrument services. (PDF 42 KB) [file 12014_2014_91_MOESM6_ESM.pdf]

Table S3 Summary of main experimental differences among the three assessment studies and major instrument services

Procedure	Description	Study I	Study III	Study II
Depletion	Date	18-21 Nov 2012	21-23 Feb 2013	15-17 Apr 2013
	Operator	JM	JM	JM
	Column lot	20093648	20093648 & SLBD7383	3315179
	HPLC machine	HPLC-LC00001	HPLC-LC00001	HPLC-LC-00001: Batch 1 & 2<br>HPLC-LC-00002: Batch 3
	HPLC service[a]	18 Nov 2012	20 & 22 Feb 2013	HPLC-LC00001: 26, 28 & 29 Mar; 15 & 17 Apr 2013<br>HPLC-LC00002: 17 Apr 2013
Digestion	Date	26-27 Nov 2012	28 Feb - 01 Mar 2013	23-24 Apr 2013
	Operator	DAS	JM	DAS/JM
Solid-phase extraction	Date	3-4 Dec 2012	03-04 Mar 2013	25-26 Apr 2013
	Operator	DAS	JM	DAS/JM
MS analysis	Date	11 Dec 2012 - 9 Jan 2013	6-10 Mar 2013	29 Apr – 5 May 2013
	Operator	JT	DAS	DAS
	SIS lot	Lot 1	Lot 1	Lot 2
	RP column lot	Not Recorded	S/N USCGC02709	S/N USCGC02348
	RP HPLC service in Year 2013	HPLC Flush (17 Jan), analytical column change (14 Feb, 21 & 25 Mar), B side check valve replaced (15 Feb), A side pump head replaced (19 Feb), B side pump head replaced (4 Mar), and PM service on LC unit (11 Mar).		
	MS service	Hexa bore capillary change (18 Dec 2012, 6 Feb 2013, 4 Mar 2013) and Ion funnels cleaned & PM on MS (11 Mar 2013).		
	Data curation	JT	DAS	DAS

[a]Changed depletion column, purge valve frit, 2 inline filters, and needle seat.
